# Unmasking Amoebiasis: An Unexpected Cause of Colitis in a Non-endemic Region

**DOI:** 10.7759/cureus.86877

**Published:** 2025-06-27

**Authors:** Vikash S Sagar, Nauman Nauman, Gayathri Jayakumar, Bismah Kazi

**Affiliations:** 1 Gastroenterology, Frimley Park Hospital, Camberley, GBR

**Keywords:** amoebiasis, amoebic colitis, crohn’s disease (cd), diarrhoea, entamoeba histolytica infection, gastrointestinal, inflammatory bowel disease, trophozoites, ulcerative colitis, colonoscopy

## Abstract

Amoebic colitis is a common mimic of inflammatory bowel disease (IBD), primarily encountered in developing countries. We present a case of a 73-year-old British male with no travel history to any amoebic endemic regions, who presented with a three-month history of per rectal (PR) bleeding, diarrhoea, and a positive faecal immunochemical test (FIT). Prior to this, he reported no history of experiencing any gastrointestinal symptoms. Colonoscopy revealed patchy pan-colitis, most marked in the ascending colon, and histology confirmed chronic inflammation. A diagnosis of IBD was made, and the patient was started on corticosteroids and 5-aminosalicylates (5-ASA). He subsequently presented to the emergency department (ED) with worsening symptoms and rising inflammatory markers. Flexible sigmoidoscopy showed progression of inflammation, and despite treatment with intravenous corticosteroids and infliximab, a tumour necrosis factor-alpha (TNF-α) inhibitor, there was no improvement. Cross-sectional imaging performed due to new-onset breathlessness during this admission identified multiple hepatic abscesses and a superior mesenteric vein thrombus. Immunosuppression was stopped, and broad-spectrum antibiotics were initiated. Liver biopsy showed inflammatory cells, but no microorganisms were seen on cultures.

Due to treatment-refractory colitis and contraindications to further immunosuppression, the patient underwent a laparoscopic subtotal colectomy with end ileostomy. Histology of the resected bowel was not in keeping with IBD, prompting re-evaluation of the initial biopsies taken from the colonoscopy at first presentation. On re-examination, amoebic-like trophozoites were seen, and the diagnosis of amoebic colitis was confirmed following review by a tertiary centre for specialist infectious disease and gastrointestinal pathology. This case highlights the need for a broad differential when managing treatment-refractory colitis, particularly in non-endemic regions, where the index of suspicion for amoebic colitis is low, and the risk of misdiagnosis is high.

## Introduction

*Entamoeba histolytica (E. Histolytica)* is the causative pathogenic protozoan for amoebic colitis [1]. Amoebiasis infections are endemic to the tropical regions of the developing world and are primarily transmitted via the faeco-oral route, as well as through sexual contact. In developed countries, amoebiasis is typically seen in tourists who travel to endemic regions, immigrants, and sexually active homosexual men [1,2].

The clinical presentation of amoebic colitis is broad and may include cramping abdominal pain, diarrhoea (watery and/or bloody), weight loss, and fever. Extra-intestinal manifestations occur via haematogenous spread to other organ systems such as the liver, brain, and lungs [3].

The differential diagnosis for* E. histolytica* intestinal amoebiasis includes bacterial pathogens such as *Shigella*, *Escherichia coli*, *Salmonella*, *Campylobacter*, and *Clostridioides difficile*. These can be differentiated based on culture results or molecular diagnostic assays. Non-infective differentials include inflammatory bowel disease (IBD) and ischaemic bowel disease [4]. Other differentials that should be considered include atypical infections such as intestinal tuberculosis and cytomegalovirus (CMV) colitis, especially in immunosuppressed populations, and collagen vascular disorders such as lupus enteritis and Behçet disease [2,4].

The clinical and endoscopic similarities between amoebic colitis and IBD can prove to be a diagnostic challenge, especially in non-endemic regions, where the index of suspicion is low. Here, we present a case of intestinal amoebiasis persistently treated as IBD, resulting in life-altering consequences.

## Case presentation

**Figure 1:**
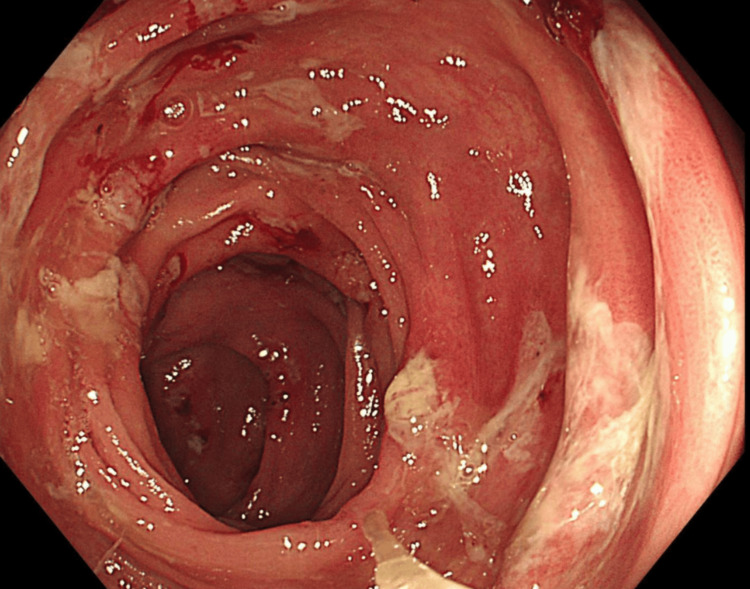
Colonoscopy of the ascending colon showing mucosal oedema, ulceration, and exudates.

A 73-year-old British male was referred via the two-week wait pathway in November 2023 after reporting a three-month history of diarrhoea, per rectal bleeding, and abdominal pain. He reported no prior history of gastrointestinal symptoms. He had no significant past medical history, and aside from travel to Greece five months prior, he had not travelled outside the United Kingdom (UK) in over 15 years. At the time of referral, faecal immunochemical test (FIT) was markedly elevated at >200 µg/g, and initial stool culture and *Clostridium difficile *toxin were negative. Specifically, stool microscopy for ova, cysts, and parasites (OCP) was not done. Outpatient colonoscopy revealed patchy pan-colitis, most marked in the right colon, with mucosal ulceration, oedema, and loss of vascular pattern (Figure 1). Based on a presumed diagnosis of an index presentation of IBD, he was started on corticosteroids and 5-aminosalicylates (5-ASA). Histology of biopsies taken during colonoscopy later showed right-sided patchy, active, chronic colitis with cryptitis and a single crypt abscess, with normal left-sided colonic biopsies and no microorganisms. Based on symptoms, colonoscopy, and histology findings, an initial diagnosis of Crohn’s colitis was made.

**Figure 2:**
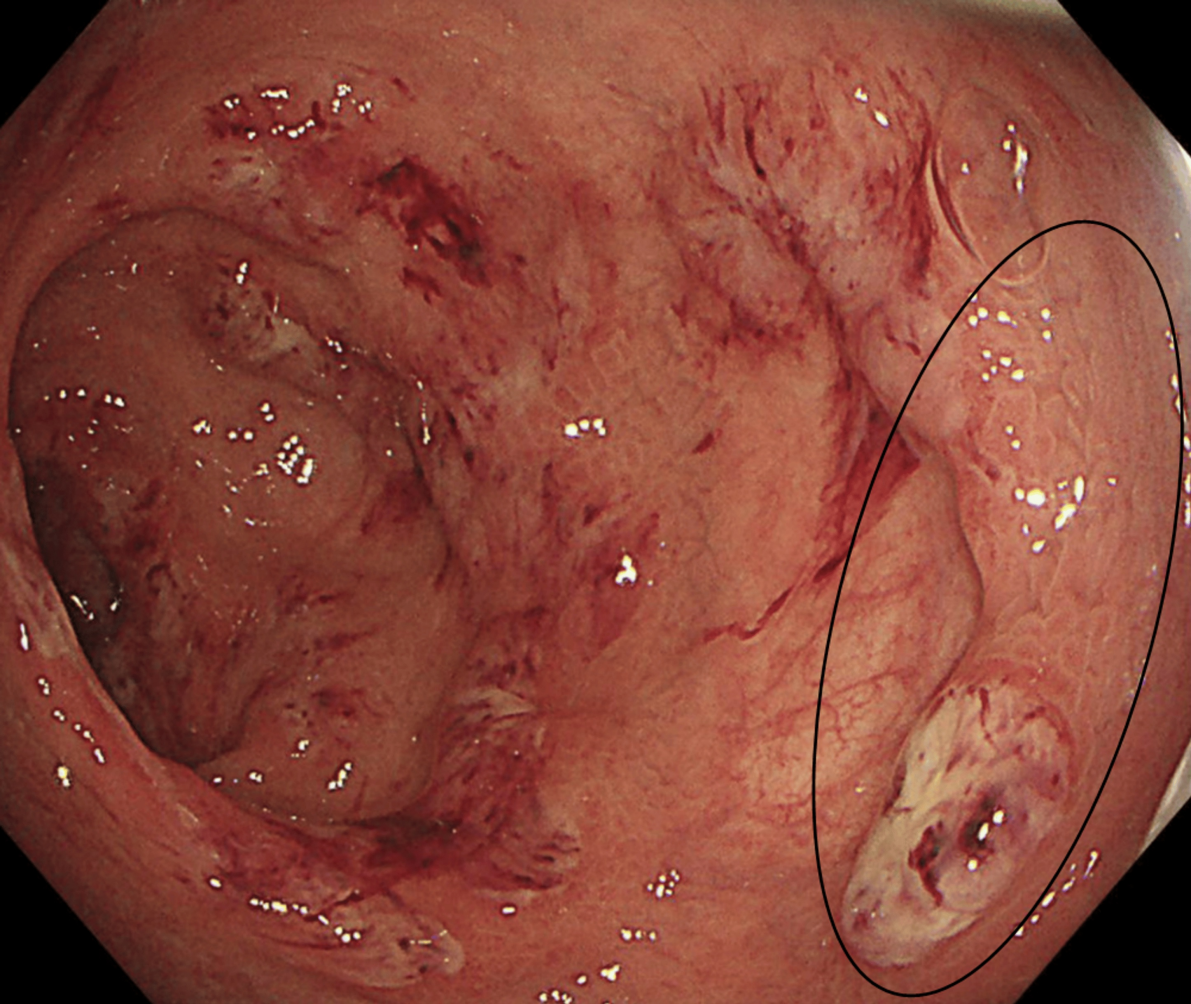
Flexible sigmoidoscopy of the rectosigmoid colon showing worsening of mucosal inflammation. Pseudopolyp (oval).

He presented to the emergency department (ED) one month later with worsening symptoms of bloody diarrhoea and abdominal pain. Initial laboratory investigations revealed a C-reactive protein (CRP) level of 12 mg/L and a white blood cell (WBC) count of 20.1 (x10^9^/L). Admission diagnosis was a flare of Crohn’s colitis, and he was commenced on intravenous corticosteroids and broad-spectrum antibiotics. Flexible sigmoidoscopy showed worsening of inflammation, now also extensively affecting the left colon (Figure 2). Due to clinical deterioration and worsening of biochemical and endoscopic findings, treatment was escalated to infliximab, a tumour necrosis factor alpha (TNF-α) inhibitor. His inflammatory markers remained elevated, but despite this, he reported mild improvement in his symptoms and was discharged with early outpatient gastroenterology review.

**Figure 3:**
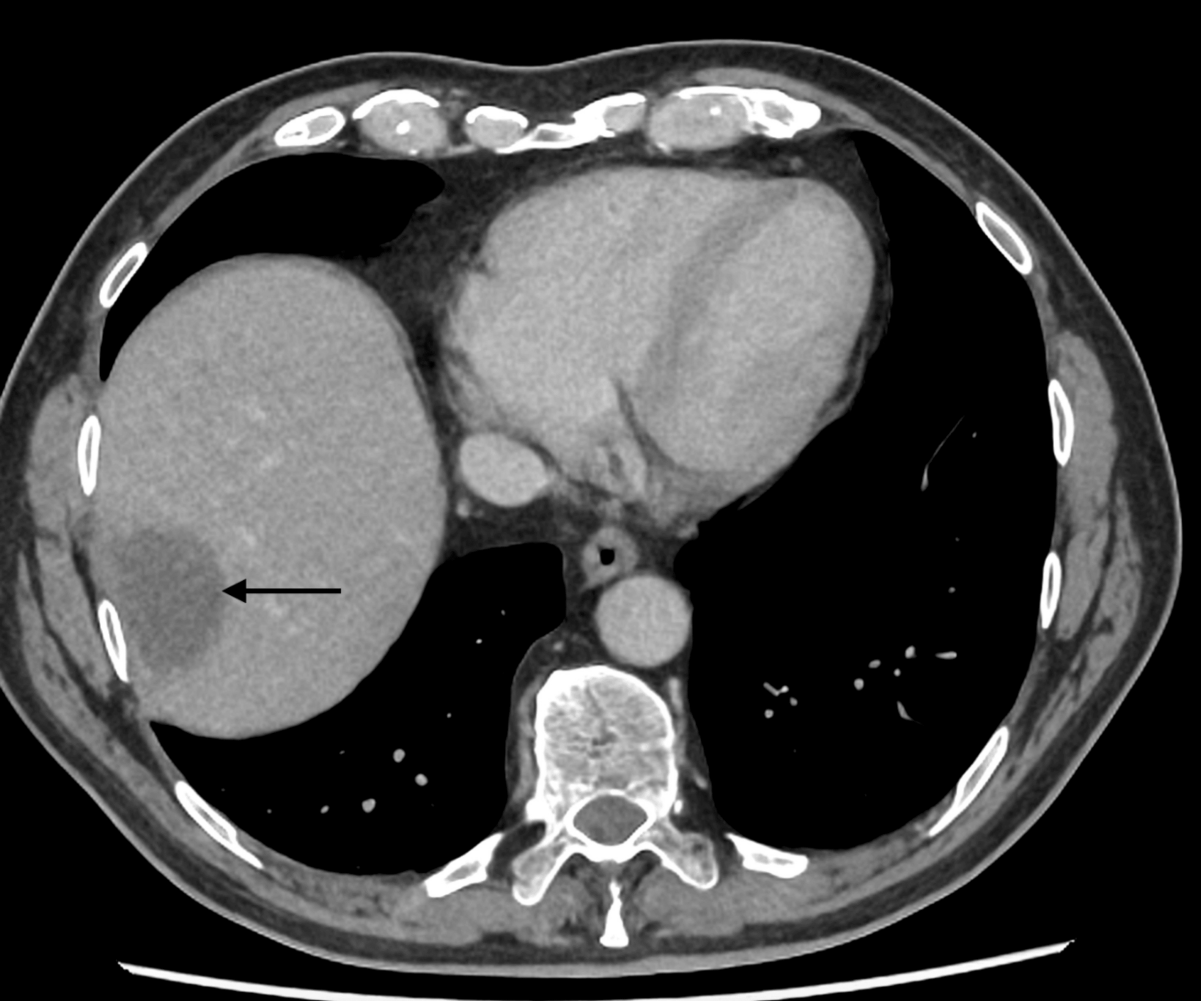
CT of the abdomen showing a hypoattenuating lesion within the right lobe of the liver in keeping with a hepatic abscess.

**Figure 4:**
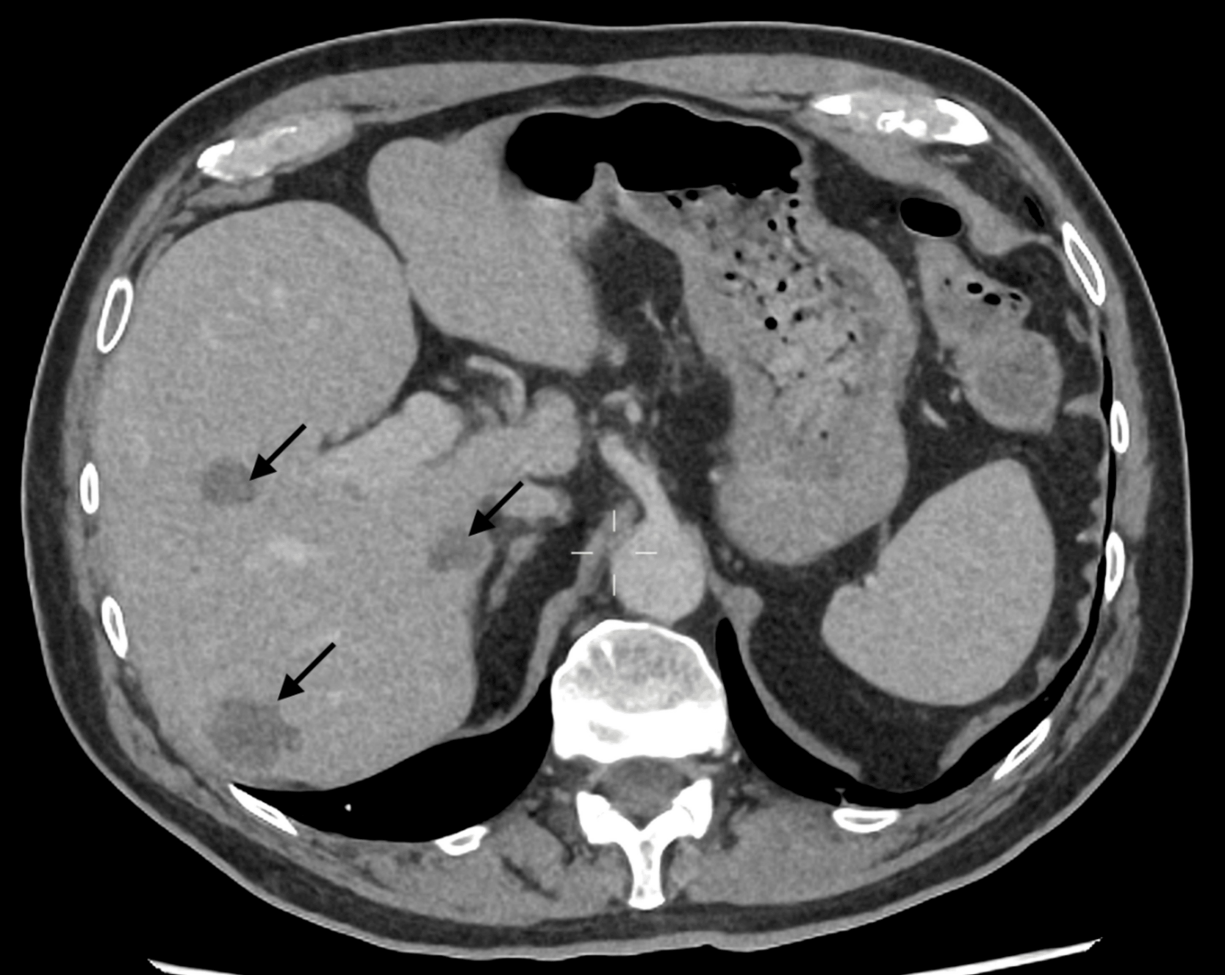
CT of the abdomen showing multiple hypoattenuating hepatic abscesses (arrows).

He re-presented to the ED nine days later with worsening bloody diarrhoea (bowels opening >10x/day), abdominal pain, and mild dyspnoea with pleuritic chest pain. Physical examination revealed mild generalised abdominal tenderness, tachypnoea with a respiratory rate of 30 breaths per minute, saturations were 97% on room air, and he was tachycardic at 114 beats per minute, but afebrile. Admission investigations showed worsening of inflammatory markers, and he was again initiated on intravenous broad-spectrum antibiotics and corticosteroids. Arterial blood gas (ABG) showed a mild respiratory alkalosis. Due to his dyspnoea and pleuritic chest pain, with an elevated respiratory rate and tachycardia, a computed tomography pulmonary angiography (CT-PA) was performed to exclude pulmonary embolism (PE), given the increased pro-thrombotic state and increased venous thromboembolic (VTE) risk associated with a presumed IBD flare. The CTPA excluded PE but incidentally revealed multiple hypoattenuating lesions scattered throughout the liver in keeping with hepatic abscesses, the largest being 4.0 centimetres, in the posterolateral aspect of the right lobe of the liver, with an enhancing rim and multiple internal septations (Figures 3, 4). Incidentally, a superior mesenteric vein (SMV) thrombus was also seen. The report also mentioned a small right-sided pleural effusion with associated compressive atelectasis. The large hepatic abscess had a compressive effect on the right hemidiaphragm, also leading to pleurisy and small pleural effusion, accounting for the patient’s breathlessness. Repeat flexible sigmoidoscopy again demonstrated worsening of colonic inflammation. Liver biopsy showed a single short core of liver tissue, which was largely replaced by necrotic debris containing a mixed inflammatory cell infiltrate consistent with an abscess; however, no amoebic organisms were seen, and microbiology cultures were negative for any organisms. The case was discussed at the IBD multi-disciplinary team (MDT) meeting. The consensus was that the multiple hepatic abscesses and SMV thrombus were secondary to uncontrolled colonic inflammation in the setting of active Crohn's disease, and given that further immunosuppression was now contraindicated due to the presence of abscesses, and previous anti-tumour necrosis factor therapy had failed, a sub-total colectomy was recommended. He was also started on low molecular weight heparin (LMWH) for the management of SMV thrombus.

**Figure 5:**
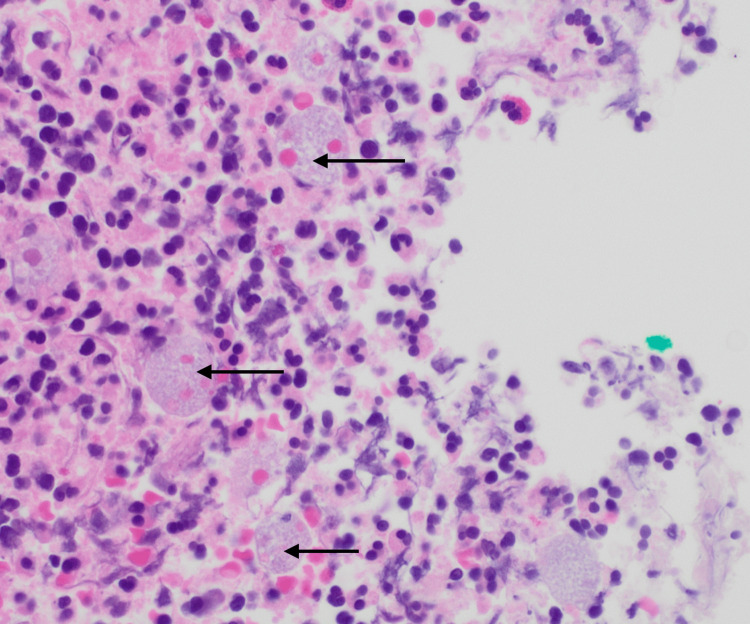
Large bowel mucosa with focal ulceration and Entamoeba histolytica trophozoites (arrows) showing erythrophagocytosis (hematoxylin and eosin stain, 40x).

**Figure 6:**
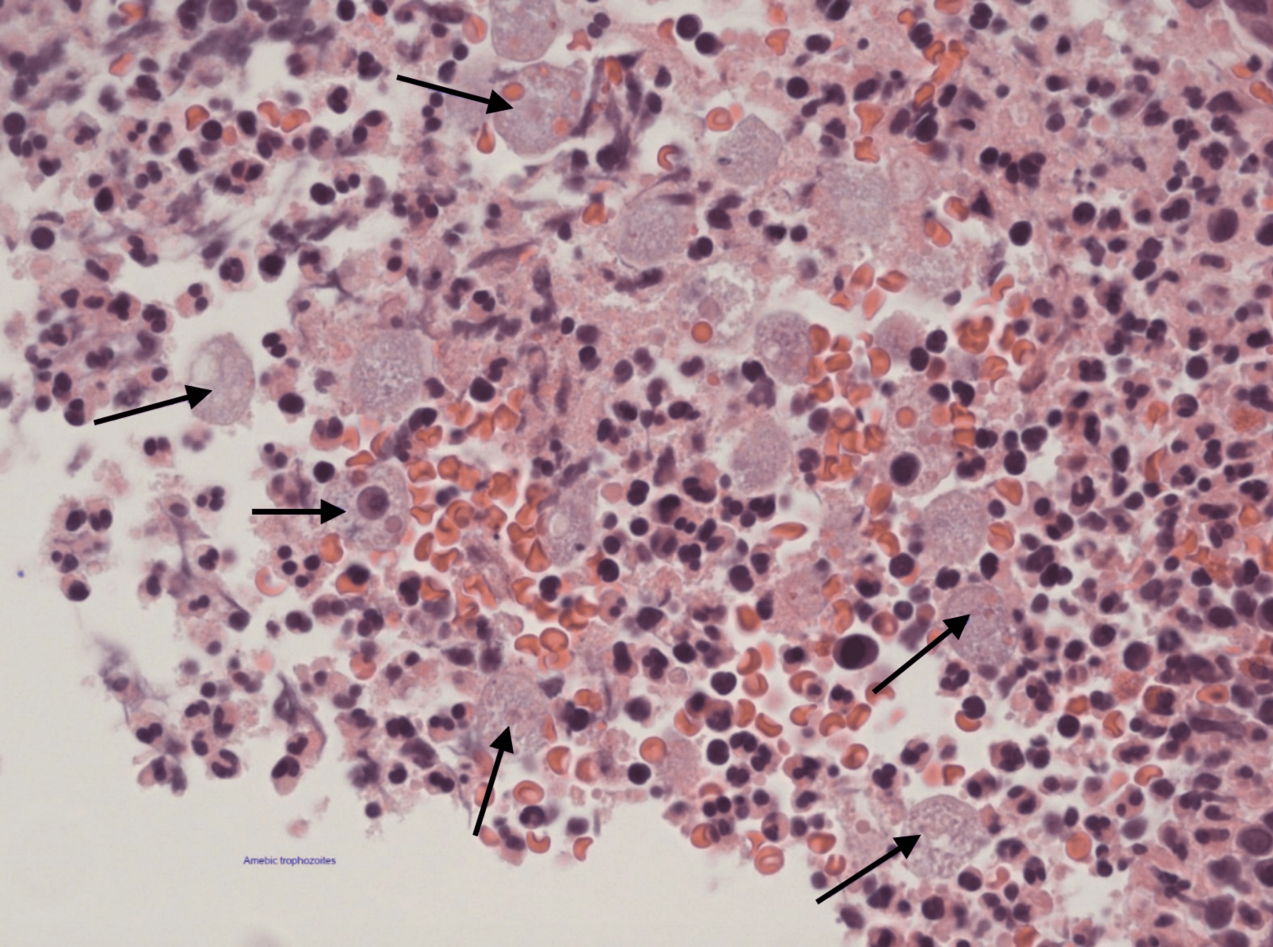
Large bowel mucosa showing amoebic trophozoites (arrows).

He underwent an uncomplicated laparoscopic subtotal colectomy with an end ileostomy. Macroscopic examination of the resected colon showed punched-out ulcers along the entire length of the colon with no evidence of cobblestoning, fissuring or stricturing disease. Histology showed scattered, discrete ulcers throughout the colon with unremarkable intervening large bowel mucosa. There was no convincing stigmata of IBD, specifically there was no cryptitis or significant crypt abscess formation, no transmural or granulomatous inflammation, and no ischaemia or microorganisms were identified. The lack of histological or macroscopic evidence of IBD prompted the histopathology team to re-review the biopsies from the initial colonoscopy from November 2023. On re-examination, a small, subtle collection of cells morphologically resembling amoebic trophozoites was identified within the ulcer slough of the caecal sample. These samples were sent to a tertiary centre for a specialist infectious disease and gastrointestinal pathology opinion. Specialist review concluded that there were unequivocal amoebic trophozoites seen in the caecal biopsies from the initial colonoscopy (Figures 5, 6); however, these were not visualised in the subtotal colectomy specimen. This may be explained by the use of anti-microbial therapy prior to surgery. There was insufficient histopathological evidence to suggest a second, co-existent inflammatory pathology such as IBD.

By this time, the patient had received a two-week course of metronidazole as part of the hospital’s local broad-spectrum antibiotics protocol and repeat interval CT of the abdomen and pelvis (CT-AP) showed resolution of the hepatic abscesses and SMV thrombus. He continued to have gastroenterology and colorectal follow-up after discharge from the hospital with complete resolution of symptoms and normalisation of laboratory investigations at six months post surgery (Table 1).

**Table 1:** Trend of investigations at first admission, one week post immunosuppression with infliximab and corticosteroids, five days postoperative colectomy and two weeks of metronidazole treatment, and six months follow-up. WBC: white blood cell count; CRP: C-reactive protein; ALT: alanine transaminase; ALP: alkaline phosphatase; FCP: faecal calprotectin.

Investigations	First admission	1-week post infliximab and steroids	5 days post colectomy and 2 weeks of metronidazole	6 months follow-up post discharge	Reference range
Haemoglobin	141	122	106	148	130-180 g/L
WBC	20.1	19.9	11.6	8.8	4-11 x 109/L
Platelet	307	362	748	247	150-450 x 109/L
CRP	12	164	11	1.7	0-5 mg/L
Albumin	34	22	27	44	35-50 g/L
ALT	17	109	26	48	0-55 U/L
ALP	102	134	109	130	30-130 U/L
Total bilirubin	8	10	4	9	0-20 umol/L
FCP	715	-	-	-	0-49.0 ug/g faeces

## Discussion

This case demonstrates the challenges and potential detrimental consequences of misdiagnosing and hence mistreating amoebic colitis as IBD in non-endemic regions where the index of suspicion is low due to a combination of low prevalence and both entities having similar clinicopathological features.

Intestinal amoebiasis typically presents with abdominal pain, diarrhoea, and dysentery, and colonoscopy findings include caecal lesions, the presence of aphthae or erosions and exudates - features also typical in IBD [5,6]. This overlap in clinical and endoscopic findings, especially in those without classical risk factors such as travel to endemic regions, immigration or high-risk sexual activity, can lead to diagnostic pitfalls.

Intestinal amoebiasis should be suspected in any case of acute or subacute diarrhoea occurring over a period of 1-3 weeks [4], whereas IBD follows a more chronic, relapsing course. Also, relevant epidemiological exposure with travel to amoebic endemic regions should raise suspicion for amoebic colitis [4]. Extra-intestinal features seen in IBD, such as arthropathies, cutaneous, ocular and hepatobiliary manifestations, can also be used to differentiate between the two clinical entities. The most common extra-intestinal feature of amoebic colitis is amoebic liver abscess (ALA), which presents with right upper abdominal pain and tenderness with fever [1]. Response to treatment can also be a key differentiator, where there should be early suspicion of an alternative diagnosis in cases of presumed IBD refractory to immunosuppressive medication [2,3].

There have been prior reports of intestinal amoebiasis being misdiagnosed as IBD [3]; however, this case is unfortunately unique due to the severity of the outcome. By the time the diagnosis of intestinal amoebiasis was eventually reached, the patient had already undergone the life-changing procedure of a subtotal colectomy and end ileostomy for presumed fulminant colitis secondary to IBD.

This highlights the need for early suspicion and reconsideration of the diagnosis in treatment-refractory cases of IBD, especially for index presentations. There needs to be increased awareness of other conditions that may mimic IBD. Failure to recognise infectious aetiologies such as amoebiasis can lead to subsequent inappropriate treatment with immunosuppression, which can lead to dissemination of infection, and increase the risk of life-threatening complications [7]. Our patient had no clinical response to corticosteroids or anti-TNF therapy. Immunosuppression likely led to the dissemination of amoebiasis, resulting in hepatic abscess formation and worsening colitis, ultimately leading to the decision for sub-total colectomy.

The diagnosis of amoebiasis can be made by multiple diagnostic modalities. Although faecal microscopy is typically used first line, it has suboptimal sensitivity at 25-60%. Other, more sensitive tools include faecal polymerase chain reaction (sensitivity >70%, specificity >90%), faecal and/or serum antigen detection (sensitivity 90% and 65% respectively in acute setting) and serology for *Entamoeba* antibodies (sensitivity 70-90%) [3,4]. Interestingly, histological visualisation of amoeba on colonic biopsy specimens is rare and hence this is not considered a routine diagnostic tool [3]. This may explain why no amoebic trophozoites were seen initially on histology but were subsequently visualised after re-examination of the original biopsies; this also required specialist centre confirmation.

Treatment for symptomatic *E. Histolytica* colitis includes systemic treatment with Nitroimidazoles such as metronidazole or tinidazole for elimination of trophozoites, followed by elimination of cysts with an intraluminal drug such as paromomycin [4,5]. Fulminant colitis additionally requires broad-spectrum antibiotic coverage for possible superimposed bacterial infection [4]. Our patient had a prolonged two-week course of metronidazole as part of the hospital’s broad-spectrum antibiotic protocol, which includes amoxicillin, metronidazole, and gentamicin, and so was inadvertently treated for amoebiasis. This can also account for the patient’s partial improvement in symptoms during his first admission, but then subsequent deterioration after antibiotics were withdrawn and immunosuppression was continued.

It is important to consider a diagnosis of intestinal amoebiasis in treatment-refractory index presentations of IBD. Misdiagnosis, inappropriate immunosuppression, and delayed antimicrobial treatment can lead to significant morbidity and mortality in these patients.

## Conclusions

Our case highlights the need for a broad differential when managing “treatment-refractory IBD” in non-endemic regions. Intestinal amoebiasis, although rare in the United Kingdom without travel or other risk factors, should be considered in any patient who fails to respond to standard IBD treatment. Early diagnosis with stool testing or serology is crucial to prevent delayed diagnosis, inappropriate immunosuppression, and subsequent potentially life-threatening complications.
